# Complications of Left Ventricular Assist Devices: A Case Study of Recurrent Bacteremia and Implications for Infection Management

**DOI:** 10.7759/cureus.74082

**Published:** 2024-11-20

**Authors:** Gabriel Velez Oquendo, Aarushi Kalra

**Affiliations:** 1 Internal Medicine, Northeast Georgia Medical Center Gainesville, Gainesville, USA

**Keywords:** bacteremia, cardiomyopathy, device therapy in heart failure, heart failure with reduced ejection fraction, left ventricular assist device(lvad)

## Abstract

Heart failure (HF) is a complex clinical condition with symptoms that result from ineffective ejection of blood due to functional or structural impairment of the heart. The most common causes of HF include ischemic heart disease, myocardial infarction (MI), hypertension, and valvular heart disease (VHD). As HF progresses to advanced stages, interventions, like left ventricular assist devices (LVADs), become essential, especially for patients ineligible for heart transplantation. However, LVADs carry substantial infection risks, particularly for bacteremia, which complicates management, patient morbidity, and patient outcomes in some cases.

Our case involves a 61-year-old male with a medical history of American College of Cardiology (ACC)/American Heart Association (AHA) stage D heart failure secondary to non-ischemic cardiomyopathy, a history of biventricular implantable cardioverter defibrillator (ICD) implantation, and an LVAD as destination therapy who experienced recurrent episodes of LVAD-associated infection. Post-implantation, he developed repeated driveline infections with Methicillin-resistant *Staphylococcus aureus* (MRSA) and *Corynebacterium*, necessitating prolonged antibiotic courses, incision and drainage, and wound care, but no device functionality issues. The patient’s non-adherence to infection control measures further complicated his clinical course, with multiple hospital admissions due to recurrent infections and associated symptoms. Psychosocial factors, including anxiety and depression, significantly impacted his adherence to driveline care, highlighting the critical role of comprehensive patient support in managing LVAD complications.

This case underscores the importance of candidate selection, patient education, and stringent infection control protocols in LVAD management. Early identification of patients at high infection risk, combined with ongoing psychosocial support, can improve outcomes and reduce complications. Given the potential for recurrent infections, enhanced screening for psychosocial issues and preventive strategies are essential for patients undergoing LVAD implantation.

## Introduction

Heart failure is when the heart cannot effectively pump blood through the circulatory system [1,2]. It is usually a condition that is caused by multiple factors, including chronically elevated blood pressure, coronary arterial disease, valvular disorders, and cardiomyopathies, among others [1,2]. One of the major causes of heart failure is cardiomyopathy, a disease of the heart that makes it difficult to pump blood to the peripheral circulation. The main types of cardiomyopathies are divided into ischemic, which is often related to coronary artery disease, and non-ischemic, which may have various etiologies [3]. It is then divided into two types: heart failure with reduced ejection fraction (HFrEF) and heart failure with preserved ejection fraction (HFpEF) [4]. Furthermore, there are four stages of heart failure that further guide management, including stage A (at risk for HF), stage B (structural heart disease without signs or symptoms of HF), stage C (structural heart disease with prior or current HF symptoms), and stage D (advanced HF requiring specialized treatments, such as mechanical circulatory support or heart transplantation) [4].

Medical treatment comprises goal-directed medical therapy (GDMT), but device therapy is sometimes required. Intracardiac devices range from implantable cardioverter-defibrillators (ICDs) and cardiac resynchronization therapy (CRT) to left ventricular assist devices (LVADs) [4]. An LVAD is a mechanical pump that supports the heart's left ventricle in pumping blood to the rest of the body. It is typically implanted in patients with severe heart failure (often referred to as stage D or end-stage heart failure) [5]. LVADs help relieve symptoms, improve quality of life, and sometimes extend survival when used as a bridge to transplant, bridge to recovery, or destination therapy, which means living with the device for the rest of the patient's life expectancy [5,6]. 

Strong indications for LVAD implantation include New York Heart Association (NYHA) class IV for 60 to 90 days, maximally tolerated medical therapy, and a certified implantable cardioverter-defibrillator if indicated. On the other hand, chronic dependence on inotropic support, left ventricular ejection fraction (LVEF) less than 25%, pulmonary capillary wedge pressure greater than or equal to 20 mmHg, and systolic BP less than or equal to 80 to 90 mmHg or cardiac index less than or equal to 2 L/min/m^2^ are other relative indications [6].

Finally, while LVADs can be beneficial, they come with potential complications, including bleeding, device thrombosis, ischemic and hemorrhagic strokes, renal insufficiency, and infections, which have been a primary cause of death in some cases [6]. They can be associated with a high risk of infection, particularly driveline infections at the site where the LVAD’s power and control cables exit the body [6]. With the increase in usage of LVADs and longer patient survival, a substantial number of patients experience adverse events and complications. LVAD-related complications can occur in up to 60% of patients by six months post-implantation, and by two years, 80% of patients experience at least one adverse event [7]. These complications can impact patient quality of life, lead to frequent hospital readmissions, and, in severe cases, contribute to mortality, as was seen in this case due to recurrent bacteremia secondary to an uncontrollable source. This poses a significant burden on the patient unless control of the infection source and lifestyle changes are implemented, as if the infection is not controlled, changing the device will not get rid of the underlying problem and could end up in recurrent infection. 

## Case presentation

This case involves a 61-year-old male with a medical history of American College of Cardiology (ACC)/American Heart Association (AHA) stage D INTERMACS II heart failure secondary to non-ischemic cardiomyopathy, history of biventricular implantable cardioverter defibrillator (ICD) implantation, and cardiac resynchronization therapy (CRT) non-responder who presented to our facility for admission before LVAD implantation, after meeting criteria for advanced heart failure therapy, without candidacy for heart transplantation due to current tobacco use disorder. 

**Figure 1:**
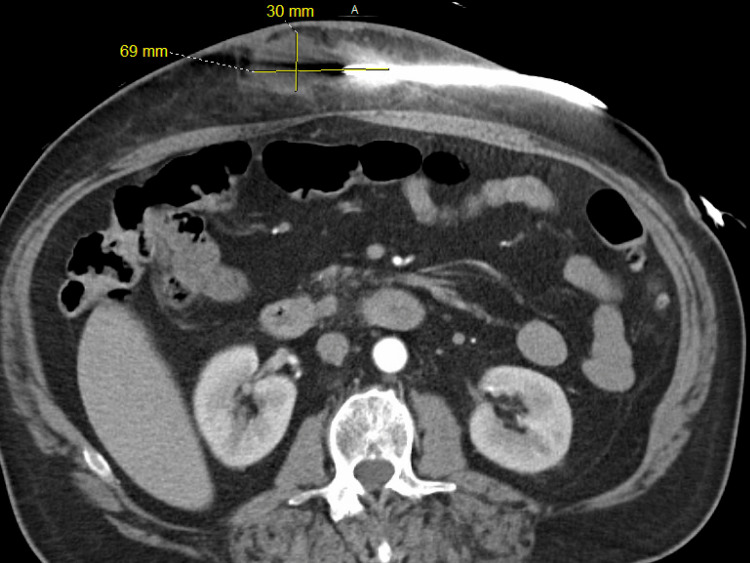
Computed tomography scan of the chest and abdomen during the initial evaluation of the LVAD driveline site shows a fluid collection (yellow lines) in the driveline site, indicating abscess formation. LVAD: left ventricular assist devices.

Management before device placement included inotropic support with milrinone 0.375 mcg/kg/min and goal-directed medical therapy with spironolactone 12.5 mg daily, Farxiga 10 mg daily, but unfortunately not on Entresto due to hypotension or Toprol-XL due to low output syndrome. After device implantation, he had expected right ventricular dysfunction leading to cardiogenic shock. The treatment concluded after discontinuation of inotropic and vasopressor support and successful resolution of cardiogenic shock with eventual discharge and close follow-up in the outpatient setting. A few days after hospital discharge, the patient presented to our emergency department (ED) after the development of burning abdominal pain with associated erythema and tan-green purulent fluid output out of the device driveline exit site. Evaluation with computed tomography (CT) of the abdomen and pelvis showed a large fluid collection (measuring 7 x 3 x 4 cm) consistent with an abscess on the right preperitoneal space beneath the tunnel incision, indicating this was the source of driveline infection (Figure 1). 

Infectious disease and thoracic surgery departments were consulted for further evaluation and recommendations. This led the surgical team to proceed with the opening of the LVAD driveline tract and the performance of incision and drainage of the subcutaneous abscess around a midportion of the driveline tract with wound bed debridement and wound vacuum-assisted closure (VAC) placement instead of pursuing antibiotic treatment only. Furthermore, the wound cultures were positive for Methicillin-resistant *Staphylococcus aureus* (MRSA), and he was started on intravenous (IV) vancomycin. He was then discharged with a PICC line to complete a prolonged course of IV antibiotics for six weeks duration and doxycycline for chronic suppression. 

Despite successful antibiotic treatment, he continuously presented to the ED with recurrent symptoms of abdominal pain and green purulent drainage from the driveline site with wound and blood cultures eventually growing *Corynebacterium* species. Because of symptoms and infection recurrence, the case was discussed at the oversight committee meeting, where they concluded to transfer the patient to a transplant center for evaluation since he had quit smoking, and his living conditions were concerning about infecting a new pump if the LVAD was exchanged. 

Unfortunately, he was denied placement on the transplant list due to inadequate social support. A few weeks afterward, the patient was admitted with the same complaints despite being on chronic doxycycline as suppression therapy. During this time, he received extensive counseling about the importance of scheduled driveline dressing changes and hygiene. Concurrently, the psychiatry team was consulted for evaluation of his underlying depression and anxiety, which continued to be ongoing and debilitating, preventing him from coping with the management of his LVAD driveline site. He continued his non-compliance with appointments with the psychiatric team as he was fearful of treatment. Despite extensive discussion with the patient regarding the severe repercussions of not managing his driveline properly, the patient continued to be non-compliant with his dressing changes and poor hygiene. As a result, he continued having multiple ED visits, with his device driveline site always in severely bad condition with soiling of the dressing and green purulent drainage underneath. 

Ultimately, he presented to the emergency department after receiving two shocks from his ICD. On admission, his devices were interrogated, showing two episodes of delivered shock due to non-sustained ventricular tachycardia not terminated by anti-tachycardia pacing. This time, the echocardiographic evaluation showed a mobile echo density on the ICD wires present in the right atrium. Consequently, the cardiac electrophysiology team was then consulted to evaluate the ICD device and lead extraction, but he opted out of any invasive procedures given the high risks. 

Eventually, the palliative medical team discussed with the patient, which concluded in a change of code status to do not resuscitate and deactivation of his ICD, which ended in the patient passing from cardiopulmonary arrest. 

## Discussion

LVAD-associated infections have a significantly higher one-year mortality rate compared to those without infections, with a prospective study showing a 22% overall infection rate of LVADs, highlighting the severe implications of bacteremia in this population [8,9]. In the REMATCH trial, where these devices were evaluated for destination therapy, 42% of the patients developed sepsis within one year of implantation [10]. Infections often involve the LVAD percutaneous driveline site due to the transcutaneous pathway of the driveline, and the chronic traumatic conditions are the triggers for infections but also involve the pump pocket, the pump interior, and the bloodstream, with multiple sites often infected [10].

Despite the high incidence and potential increase in morbidity and mortality associated with LVADs, the epidemiology has not been well described. Identifying patients at higher risk of infection through pre-operative evaluation can improve patient outcomes by selecting candidates more likely to benefit from LVAD support without facing high infection risk. Researchers have identified factors like diabetes, prior infection history, older age, malnutrition, and renal dysfunction as risk factors for post-implantation infections as they contribute to morbidity and complications in heart failure care [10,11].

Among LVAD-specific problems, driveline site infections are the predominant adverse events experienced by patients, frequently leading to severe complications and recurrent hospital readmissions, therefore representing a major risk factor for late mortality and posing considerable structural and financial challenges to patients and caregivers [12]. This patient’s risk factors, including his depression disorder, contributed to his non-compliance with his wound care and outpatient office visit follow-up. This, in turn, complicated his case because even though he completed antibiotic treatment multiple times for infection source control, he never implemented prevention maneuvers. By improving guidelines for prevention and early intervention strategies, ongoing research can help reduce hospital expenses and improve cost-effectiveness in managing heart failure patients with LVADs. By implementing risk scores based on patient factors such as immune status, nutritional health, and co-existing conditions, clinicians can take pre-emptive steps for higher-risk patients, such as close monitoring and early intervention [12].

A recently published expert consensus reviewed the existing knowledge on the management of patients on long-term mechanical circulatory support with the aim of an improved quality of life. In this document, pre-operative patient selection, patient risk factors, surgical preparations, end-of-life care, and the need for a dedicated postoperative multidisciplinary care approach are highlighted [13]. In particular, it is crucial for the outpatient care setting to involve a multidisciplinary approach, such as specialized physicians, nurses and LVAD coordinators, rehabilitation centers, general practitioners, outpatient LVAD specialists, and outpatient nursing services and relatives [13].

This document also emphasizes prevention and early treatment after the perioperative period, irrespective of surgical tunneling techniques used for LVAD implantation [13]. Before discharge into rehabilitation, the patient and the relatives should be trained on the wound dressing change procedure wherever possible [13]. Dressing changes are then monitored and re-trained upon the next appointment in the outpatient care clinic, including wound staging, to guide further management [13].

This case highlights the severe and recurrent complications associated with LVAD therapy, particularly LVAD-associated bacteremia. It underscores the importance of patient adherence, rigorous infection control, and comprehensive support in improving outcomes. The patient’s course illustrates how chronic infection, compounded by psychosocial challenges, can complicate the management of LVADs and ultimately affect survival. Recurrent LVAD infections, especially bacteremia caused by antibiotic-resistant pathogens, present significant challenges to treatment and increase the risk of both morbidity and mortality. Patient selection is crucial and involves multidisciplinary evaluation, including assessment of psychosocial factors, adherence potential, and caregiver support, as LVAD management requires substantial lifestyle adjustments and ongoing care.

## Conclusions

This case highlights the critical need for meticulous infection management, patient education, and comprehensive psychosocial support for individuals with LVADs. Recurrent LVAD-associated bacteremia not only affects patient survival and quality of life but also imposes a considerable burden on healthcare resources due to frequent hospitalizations and complex care needs. As for this patient, he was counseled and taught about regular and proper wound care, which he unfortunately did not follow. Moreover, he was counseled on timely follow-ups with psychiatry/social work to improve his self-care. Still, he did not proceed despite multiple admissions for infection and the counseling provided.

Prioritizing early identification of infection risk factors and implementing preventive strategies tailored to each patient's psychosocial and clinical profile can mitigate these risks, including possible home health services for wound care management. Enhanced interdisciplinary approaches involving infectious disease, cardiology, and mental health support are essential to improving patient adherence, reducing infection rates, and ultimately optimizing outcomes for LVAD recipients. Further research is warranted to establish evidence-based protocols for infection prevention, such as the establishment of a team that provides care at home and psychosocial support in a periodic manner.
